# Serum HBsAg and HBcrAg is associated with inflammation in HBeAg-positive chronic hepatitis B patients

**DOI:** 10.3389/fcimb.2023.1083912

**Published:** 2023-03-31

**Authors:** Jing Zhao, Dandan Bian, Hao Liao, Yang Wang, Yan Ren, Yingying Jiang, Shuang Liu, Xinyue Chen, Zhongjie Hu, Zhongping Duan, Fengmin Lu, Sujun Zheng

**Affiliations:** ^1^ Liver Disease Center, Beijing YouAn Hospital, Capital Medical University, Beijing, China; ^2^ Department of Infectious Diseases, Electric Power Teaching Hospital, Capital Medical University, Beijing, China; ^3^ Department of Microbiology and Infectious Disease Center, Peking University Health Science Center, Beijing, China; ^4^ Intervention and Cell Therapy Center, Peking University Shenzhen Hospital, Shenzhen Peking University-The Hong Kong University of Science and Technology Medical Center, Shenzhen, Guangdong, China

**Keywords:** chronic hepatitis B, HBsAg, HBcrAg, inflammation, nucleos(t)ide analogues

## Abstract

**Backgrounds & aims:**

Liver inflammation is the main risk factor for developing liver fibrosis, cirrhosis, and even hepatocellular carcinoma in chronic hepatitis B (CHB) patients. To replace biopsy, additional non-invasive biomarkers to diagnose and grade liver necroinflammation are urgently required in clinical practice.

**Method:**

Ninety-four CHB patients, including 74 HBeAg-positive and 20 HBeAg-negative patients, were enrolled and started entecavir or adefovir therapy. Serum HBV RNA, HBV DNA, HBsAg, hepatitis B core-related antigen (HBcrAg), ALT and AST levels, as well as intrahepatic HBV DNA and cccDNA were measured at baseline and during treatment. Liver inflammation was assessed at baseline and month 60 by liver biopsy. Inflammation regression was defined as a ≥1-grade decrease according to the Scheuer scoring system.

**Results:**

In HBeAg-positive CHB patients, at baseline, serum HBsAg and HBcrAg levels negatively correlated with inflammation grade, while ALT and AST levels positively correlated with inflammation grade. AST plus HBsAg exhibited excellent diagnostic ability for significant inflammation with an AUROC of 0.896. After 60 months of antiviral treatment, almost all the patients’ liver inflammation ameliorated to G1, and no patients had inflammation progression.

**Conclusion:**

Besides ALT and AST, serum HBsAg and HBcrAg correlated with inflammation grade in HBeAg-positive CHB patients before NAs treatment. Moreover, the combination of HBsAg and AST exhibited excellent diagnostic ability for significant inflammation.

## Introduction

Hepatitis B virus (HBV) infection is a major public health issue affecting about 250 million individuals globally (Schweitzer et al., 2015; World Health Organization, 2017). As a noncytopathic virus, HBV leads to hepatocellular injuries mediating by the host’s immune response to the inflammatory damage in hepatocytes (Liaw et al., 1983; Perrillo, 2001; Liaw, 2003). Chronic hepatic inflammation not only hinders the body from clearing HBV but also promotes the development of liver fibrosis, cirrhosis, and even hepatocellular carcinoma (HCC) (Revill et al., 2019). Thus, it is essential to evaluate the grade of liver inflammation early and effectively reverse its progression in chronic hepatitis B (CHB) patients.

Liver biopsy is still considered the gold standard to evaluate inflammation, but the invasiveness limited its application (Sarin et al., 2016). Alanine aminotransferase (ALT) and aspartate aminotransferase (AST) are indicators of liver injury and widely used to reflect liver necroinflammation. Nevertheless, there are limitations in their accuracy in evaluating inflammation in CHB patients (Nguyen et al., 2015). Additional non-invasive biomarkers to diagnose and grade liver necroinflammation are urgently required in clinical practice.

In this study, we aimed to assess the correlation of serum and intrahepatic HBV markers, including serum HBsAg, HBcrAg, HBV DNA and HBV RNA, as well as intrahepatic HBV DNA and cccDNA, with the degree of liver inflammation according to Scheuer scoring system (Scheuer, 1991) in CHB patients before and during nucleos(t)ide analogues (NAs) therapy. Furthermore, we analyzed the performances of these makers in diagnosing significant liver necroinflammation before NAs treatment, and their dynamic changes during NAs treatment.

## Material and methods

### Patients and study design

This study was conducted using a cohort of 94 CHB patients receiving NAs monotherapy, who were prospectively recruited from Beijing YouAn Hospital, Capital Medical University (Beijing, China) between June 2007 and July 2008. CHB patients who were diagnosed by the American Association for the Study of Liver Diseases guideline (Terrault et al., 2016) were enrolled if they aged ≥ 16 years and were treat-naïve. The exclusion criteria were as follows: (a) co−infection with hepatitis C, hepatitis D virus, or human immunodeficiency viruses; (b) existence of the alcoholic liver disease or autoimmune liver disease; (c) with decompensated cirrhosis or HCC; (d) with a history of immunosuppressive therapy or organ transplantation; (e) pregnant or breastfeeding women.

Once recruitment, patients were given entecavir (ETV) or adefovir (ADV) and followed up. At each follow-up, serum samples were collected for HBV DNA quantification and liver function tests. The remaining serum specimens were stored at -80°C for subsequent research. At enrollment and Month 60, percutaneous liver biopsies were performed to evaluate the histology. With cryopreserved serum samples, HBsAg and HBcrAg levels at baseline, months 6 and 60, as well as HBV RNA levels at baseline, months 6, 12, 24, 36, 48, and 60 were retrospectively quantified.

This study followed the Declaration of Helsinki and was approved by the Institutional Review Board of Beijing YouAn Hospital, Capital Medical University (Beijing, China). All subjects provided written informed consent.

### Assays for serum HBsAg, HBcrAg, HBV RNA and HBV DNA

Serum HBsAg was quantified by Elecsys HBsAg II Quant reagent kits (Roche Diagnostics) with a lower limit of detection (LLD) of 0.05 IU/mL. Quantitative levels of HBcrAg were determined by chemiluminescent enzyme immunoassay in an automated analyzer system (Fujirebio Inc., Tokyo, Japan) with the LLD of 1,000 IU/mL and a linear range of 3-7 log IU/mL. Serum HBV RNA was isolated with the nucleic acid extraction or purification kit (Sansure Biotech, Changsha, China) and treated with DNase I (Thermo Fisher Scientific, Waltham, MA, USA). The LLD of the assay was 200 copies/mL. Details on HBV RNA assay can be found in **Supplementary Materials**. The serum HBV DNA level was determined using the Cobas HBV Amplicor Monitor assay (Roche Diagnostics, Pleasanton, CA, USA) with an LLD of 50 IU/mL.

### Hepatic histological evaluation

At baseline and after 60 months of NAs treatment, a percutaneous liver biopsy was performed. A minimal 18mm length of liver tissue containing at least three complete portal tracts was obtained for pathological evaluation (Colloredo et al., 2003). All liver biopsies were reviewed continuously by an experienced pathologist blinded to treatment assignment and time of biopsy. Inflammation was assessed according to the Scheuer scoring system, which is entirely based on histology results. And histologic findings of portal inflammation, interface hepatitis, and lobular inflammation are assigned a score ranging from 0 to 4 (Scheuer, 1991). G≥3 was defined as having significant inflammation. Inflammation regression was defined as a ≥1-grade decrease according to the Scheuer scoring system.

### Quantitation of intrahepatic HBV DNA and cccDNA

For DNA extraction, about 30 μm formalin fixation and paraffin embedding (FFPE) liver biopsy tissue in sections of 6 μm each were used. QIAamp FFPE DNA Mini Kit (QIAGEN, GmbH, Hilden, Germany) was used to extract DNA according to the instructions of the manufacturer. HBV rcDNA, replicative dsDNA, and ssDNA were digested using T5 Exonuclease (New England Biolabs, USA). The reaction mixture contained 100 ng extracted DNA, 0.5 µL (10 units) T5 Exonuclease, 1 µL NEBuffer 4 (10×) with Nuclease-free H2O to a final volume of 10 μL. The digestion was conducted at 37°C for 1 h, and stop the reaction with EDTA to at least 11 mM. We combined 6.42 μL of digestion product obtained in the previous step, with 7.50 μL QuantStudio™ 3D Digital PCR Master Mix, 0.06 μL of TaqMan Probe-RC-MGB (50 μM), 0.06 μL TaqMan Probe-RNAseP-VIC (50 μM), 0.24 μL primer of rc-F, 0.24 μL primer of rc-R, 0.24 μL primer of RNaseP-F and 0.24 μL primer of RNaseP-R. 15μL of this sample mix was added to each chip and loaded on ProFlex™ 2x Flat PCR System. Absolute quantification was determined with QuantStudio™ 3D Digital PCR System (Thermo Fisher Scientific Inc., Waltham, Massachusetts, USA) and analyzed using QuantStudio 3D AnalysisSuite Cloud Software. (https://china.apps.thermofisher.com/quantstudio3d/). Intrahepatic HBV cccDNA values were normalized to cell number assessed by RNase P copy number assay.

### Statistical analysis

Continuous variables were expressed as medians and ranges or means and standard deviations and categorical variables as frequencies. Differences between groups were analyzed using the student t or Mann-Whitney tests for continuous parameters and chi-square or Fisher exact tests for categorical parameters, as appropriate. The receiver operating characteristic (ROC) analysis assessed the markers’ diagnostic capacity for inflammation with the cut-off values determined using the Youden index. The 95% confidence interval of the area under the ROC curve (AUROC) was determined using a bootstrap method. The regression and Spearman correlation coefficients (r) were used to depict the correlation between the two variables. All analyses were performed with SPSS version 26.0, with a two-tailed p-value <0.05 considered statistically significant.

## Results

### Baseline characteristics of the patients

A total of 94 CHB patients who performed liver biopsies at baseline were analyzed in this study, including 74 HBeAg-positive and 20 HBeAg-negative patients. **Table 1** summarizes the characteristics of this population, which was predominantly male (n=74, 78.7%) with a median age of 35.5 years and a median BMI of 23.8. Forty-two patients had available HBV genotype data, with 71.4% (30/42) genotype C. Of the 94 patients, 47.9% (45/94) were treated with ETV, and 52.1% (49/94) were treated with ADV after recruitment. The median serum ALT and AST were 69.8 U/L and 44.1 U/L, respectively. Median levels of baseline serum HBV RNA, HBV DNA, HBsAg and HBcrAg were 5.08 log10copies/mL, 6.27 log10IU/mL, 3.52 log10IU/mL and 6.72 log10IU/mL, respectively. Median levels of intrahepatic HBV DNA and cccDNA are 6.44 and 4.73 log10 copies/10^5^ cells.

**Table 1:** Baseline characteristics of the patients.

	Total (n=94)	HBeAg(+) (n=74)	HBeAg(-) (n=20)	*P*
Age (year)	35.5(16-60)	35(16-60)	41.5(24-56)	0.15
Male/Female	74/20	58/16	16/4	1.0
BMI (Kg/m^2^)	23.8(16.2-32.9)	23.8(17.2-32.9)	23.8(16.2-31.5)	0.84
HBV Genotype †				0.67
C/others	30/12	25/9	5/3	
Treatment (n (%))				***0.03***
ETV/ADV	45/49	40/34	5/15	
ALT (U/L)	69.8(12.6-681.9)	68.5(36.1-113.0)	102.3(17.2-527.5)	0.38
AST (U/L)	44.1(10.9-358.8)	44.0(28.9-69.6)	56.2(16.0-200.6)	0.26
HBVDNA (log_10_IU/mL)	6.27(1.70-9.28)	6.71(1.99-9.28)	4.55(1.70-8.32)	***0.004***
HBVRNA (log_10_copies/mL)	5.08(1.40-8.49)	5.43(2.00-8.49)	3.71(1.40-6.10)	***<0.001***
HBV RNA/DNA ratio	0.79(0.34-1.83)	0.82(0.34-1.83)	0.69(0.38-1.61)	0.53
HBsAg (log_10_IU/mL)	3.52(-0.07-4.95)	3.77(-0.07-4.95)	3.07(1.67-3.63)	***0.006***
HBcrAg (log_10_IU/mL)	6.72(3.40-8.73)	7.32(4.84-8.73)	5.29(3.40-7.27)	***<0.001***
Intrahepatic HBV DNA (log10copies/10^5^ cell)	6.44(3.88-8.50)	6.60(4.42-8.50)	5.82(3.88-6.98)	***0.02***
Intrahepatic cccDNA (log10copies/10^5^ cell)	4.73(2.69-7.18)	4.90(2.69-7.18)	4.05(3.12-5.09)	***0.004***

†Forty-two patients with available genotype data were analyzed.

Continuous variables are expressed as medians and ranges; categorical variables are expressed as frequencies.

ALT, alanine aminotransferase; AST, aspartate aminotransferase; BMI, Body Mass Index; ETV, Entecavir; ADV, Adefovir dipivoxil; HBsAg, hepatitis B surface antigen; HBcrAg, hepatitis B core-related antigen; HBeAg, hepatitis B e antigen; cccDNA, covalently closed circular DNA.

P values <0.05 are shown in bold.

Compared to HBeAg-negative CHB patients, HBeAg-positive patients have significantly higher serum HBV DNA, HBV RNA, HBsAg and HBcrAg, as well as intrahepatic HBV DNA and cccDNA.

### Correlation of serum and intrahepatic HBV markers, ALT and AST with inflammation grade according to Scheuer scoring system

**Figure 1:**
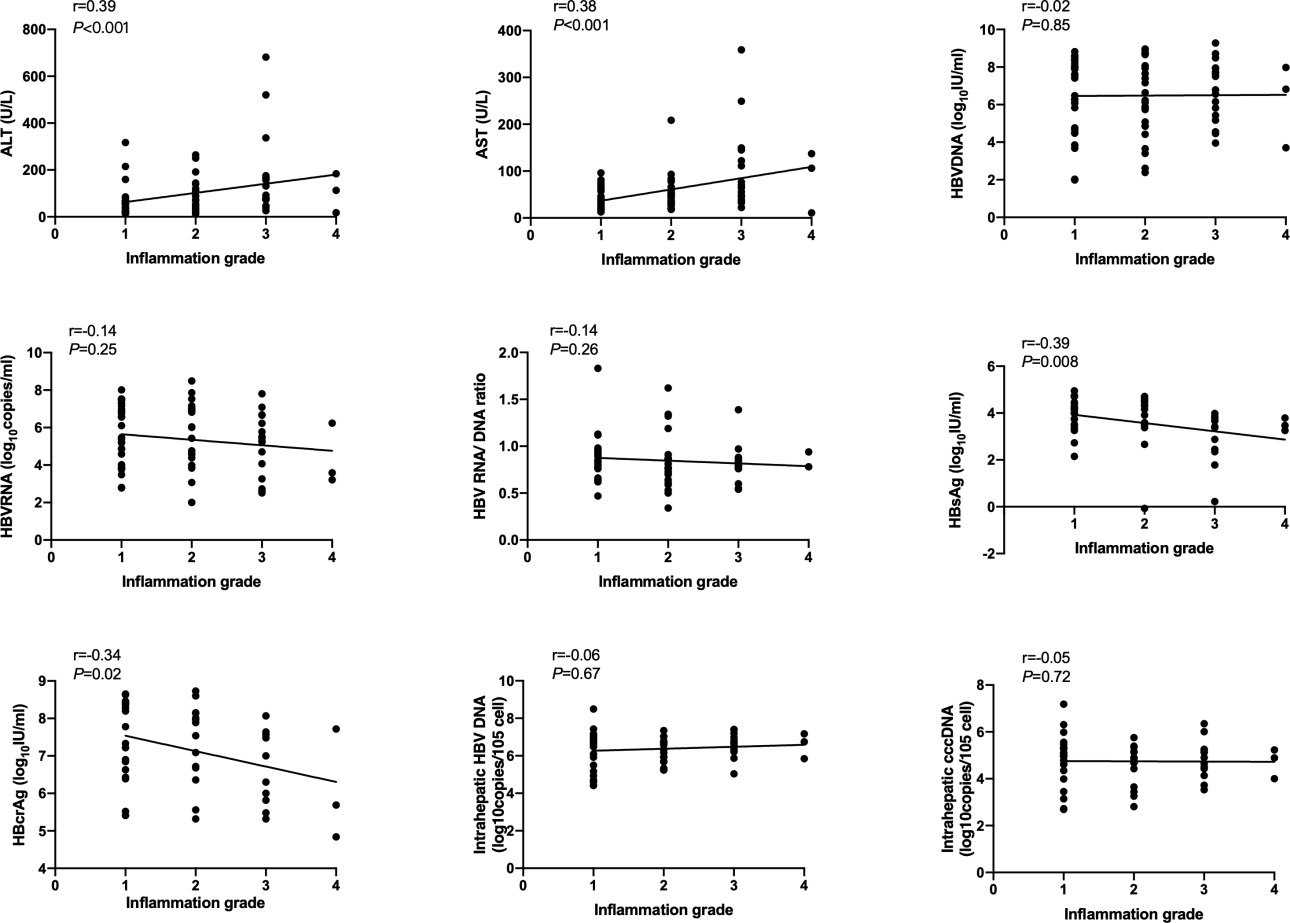
Correlation of HBV markers, ALT and AST with inflammation grade according to Scheuer scoring system in HBeAg-positive CHB patients at baseline. HBsAg, hepatitis B surface antigen; HBcrAg, hepatitis B core-related antigen.

**Figure 2:**
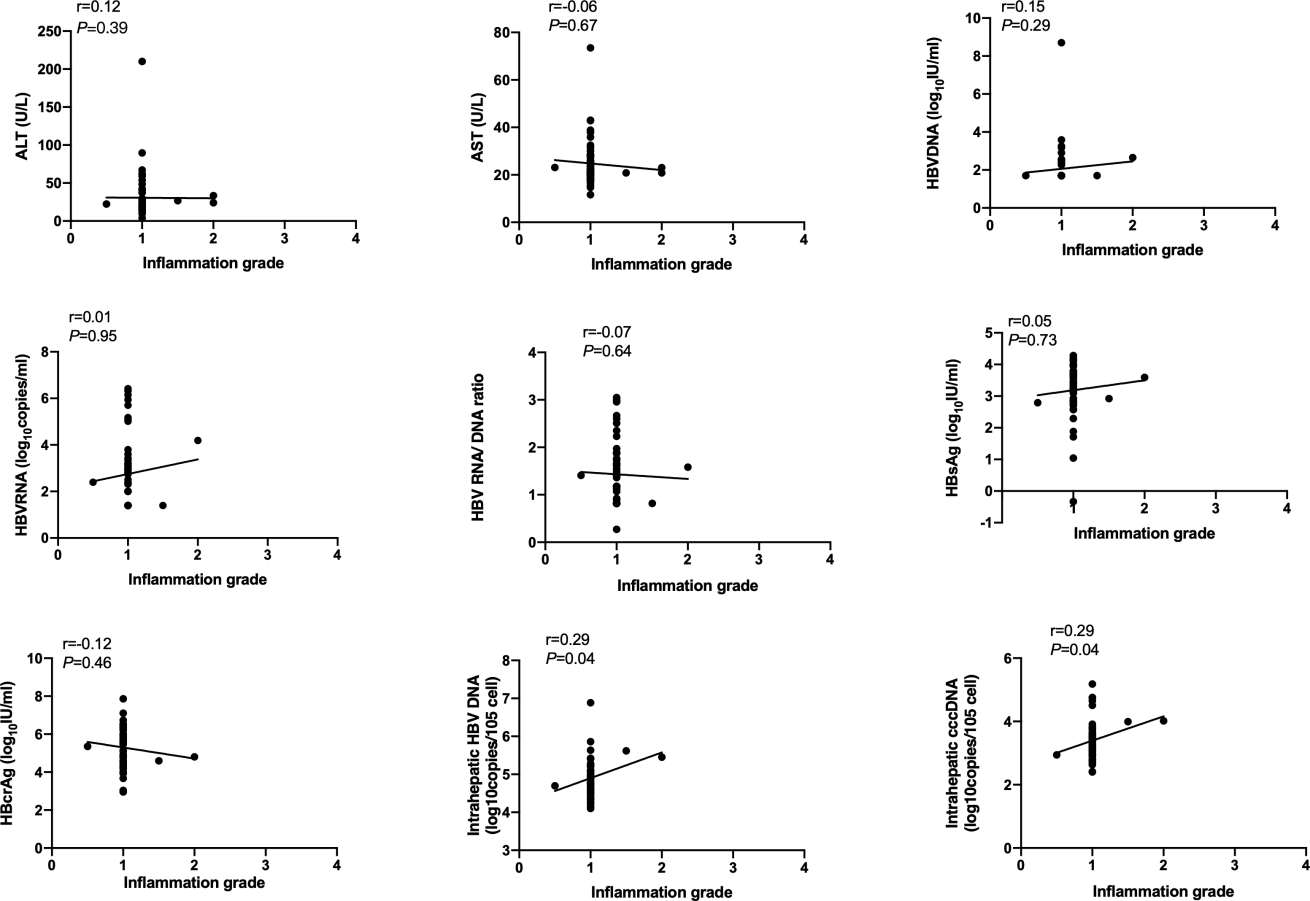
Correlation of HBV markers, ALT and AST with inflammation grade according to Scheuer scoring system in HBeAg-positive CHB patients after 60 months of NAs therapy. HBsAg, hepatitis B surface antigen; HBcrAg, hepatitis B core-related antigen.

Spearson’s correlation coefficients (r) were used to evaluate the correlation of serum and intrahepatic HBV markers, ALT and AST with inflammation grade according to the Scheuer scoring system. As shown in **Figure 1**, serum HBsAg and HBcrAg levels weakly negatively correlated with inflammation grade (r=-0.39, P=0.008; r=-0.34, P=0.02), while ALT and AST levels weakly positively correlated with inflammation grade (r=0.39, P<0.001; r=0.38, P<0.001) in HBeAg-positive patients at baseline. Nonetheless, after 60 months of NAs treatment, these correlations disappeared, and intrahepatic HBV DNA and cccDNA got a positive correlation with inflammation grade (r=0.29, P=0.04; r=0.29, P=0.04) (**Figure 2**).

In HBeAg-negative patients, all these serum and intrahepatic HBV markers, ALT and AST had no relevancy with inflammation grade neither at baseline nor after 60 months of NAs treatment (**Supplementary Figures 1, 2**).

### Factors associated with significant inflammation at baseline

**Figure 3:**
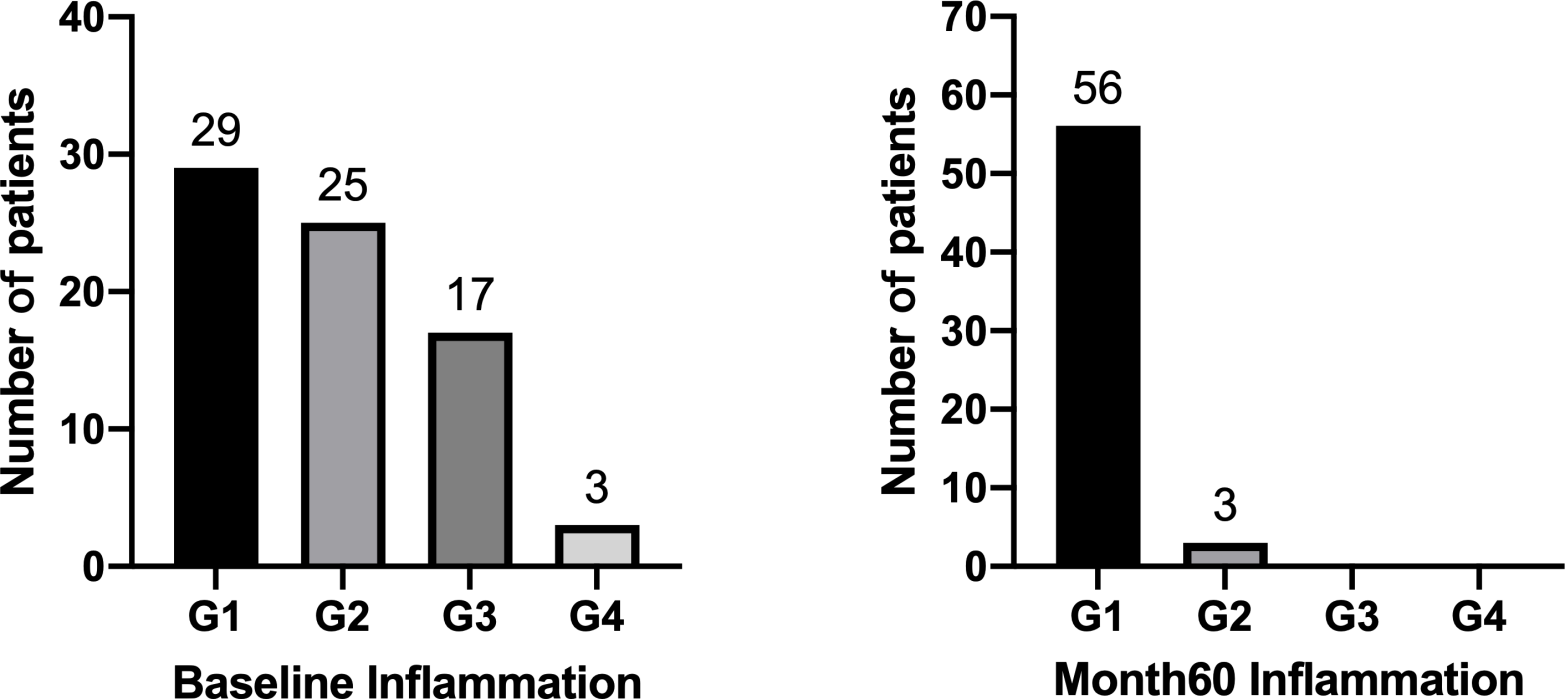
Inflammation changes after 60 months of NAs therapy in HBeAg-positive CHB patients.

At baseline, HBeAg-positive patients with G1, G2, G3 and G4 were 29(39.2%), 25(33.8%), 17(23.0%) and 3(4.1%) respectively (**Figure 3**). We defined G≥3 as significant inflammation, and the associated factors are depicted in **Table 2**. The results showed that patients with significant inflammation had lower serum HBsAg and HBcrAg, higher serum ALT and AST, as well as older age. To evaluate the abilities of these variables to diagnose significant inflammation, ROC analyses were conducted. HBsAg performed best with the AUROC of 0.784 (95%CI 0.655-0.912; P=0.002). While the AUROCs of ALT, AST, HBcrAg and age were 0.733 (95%CI 0.601-0.865; P=0.002), 0.725 (95%CI 0.586-0.865; P=0.003), 0.728 (95%CI 0.574-0.883; P=0.02) and 0.677 (95%CI 0.541-0.812; P=0.02), respectively. Moreover, the combination of these markers would further improve the performance, with AST>101(U/L) plus HBsAg<4(log10IU/mL) having the best AUROC of 0.896 (95% CI 0.802-0.990; P<0.001), of which the sensitivity and specificity are 57.1% and 100%, respectively (**Table 3**).

**Table 2:** Variables associated with significant inflammation (G ≥ 3 according to the Scheuer scoring system) in HBeAg-positive CHB patients at baseline.

	Total (n=74)	Liver inflammation grade at baseline		
		G <3 N= (54)	G ≥ 3 N= (20)	*P*
Age (year)	35(16-60)	32.5(16-53)	40(25-60)	***0.02***
Male/Female	58/16	40/14	18/2	0.21
BMI (Kg/m^2^)	23.8(17.2-32.9)	23.6(17.2-32.9)	24.6(21.9-30.1)	0.18
HBV Genotype †				0.40
C/others	25/9	18/8	7/1	
Treatment (n (%))				0.30
ETV/ADV	40/34	27/27	13/7	
ALT (U/L)	68.5(36.1-113.0)	58.9(12.6-317.3)	102.5 (17.4-681.9)	***0.04***
AST (U/L)	44.0(28.9-69.6)	36.7(12.9-208.6)	67.1(10.9-358.8)	***0.02***
HBVDNA (log_10_IU/mL)	6.71(1.99-9.28)	6.56(1.99-8.96)	6.80(3.70-9.28)	0.65
HBVRNA (log_10_copies/mL)	5.43(2.00-8.49)	5.45(2.00-8.49)	5.26(2.51-7.80)	0.23
HBV RNA/DNA ratio	0.82(0.34-1.83)	0.84(0.53-1.83)	0.78(0.34-1.19)	0.10
HBsAg (log_10_IU/mL)	3.77(-0.07-4.95)	4.12(-0.07-4.95)	3.40(0.22-3.98)	***0.02***
HBcrAg (log_10_IU/mL)	7.32(4.84-8.73)	7.66(5.32-8.77)	6.31(4.84-8.07)	***0.02***
Intrahepatic HBV DNA (log10copies/105 cell)	6.60(4.42-8.50)	6.55(4.42-8.50)	6.67(5.03-7.41)	0.19
Intrahepatic cccDNA (log10copies/105 cell)	4.90(2.69-7.18)	4.89(2.69-7.18)	4.90(3.53-6.35)	0.67

†Thirty-four patients with available genotype data were analyzed.

Continuous variables are expressed as medians and ranges; categorical variables are expressed as frequencies.

ALT, alanine aminotransferase; AST, aspartate aminotransferase; BMI, Body Mass Index; ETV, Entecavir; ADV, Adefovir dipivoxil; HBsAg, hepatitis B surface antigen; HBcrAg, hepatitis B core-related antigen; HBeAg, hepatitis B e antigen; cccDNA, covalently closed circular DNA.

P values <0.05 are shown in bold.

**Table 3:** Performance of variables to diagnose significant inflammation (G ≥ 3 according to the Scheuer scoring system) in HBeAg-positive patients at baseline.

	AUROC	*P*	Cut-off	Sensitivity (%)	Specificity (%)
Age(year)	0.677(0.541-0.812)	***0.02***	33.5	85.0	51.9
ALT (U/L)	0.733(0.601-0.865)	***0.002***	73.4	80.0	72.2
AST(U/L)	0.725(0.586-0.865)	***0.003***	101.1	40.0	98.1
HBsAg (log_10_IU/mL)	0.784(0.655-0.912)	***0.002***	3.98	100.0	57.6
HBcrAg (log_10_IU/mL)	0.728(0.574-0.883)	***0.02***	7.75	92.3	50.0
HBsAg>4(log_10_IU/mL)&HBcrAg >7(log_10_IU/mL)	0.758(0.624-0.891)	***0.006***		100.0	51.5
ALT>73(U/L) &AST>101(U/L)	0.804(0.681-0.927)	***<0.001***		40.0	98.1
ALT>73(U/L) &HBsAg<4(log10IU/mL)	0.872(0.772-0.973)	***<0.001***		78.6	84.8
AST>101(U/L) &HBsAg<4(log10IU/mL)	0.896(0.802-0.990)	***<0.001***		57.1	100.0
ALT>73(U/L) &HBcrAg<7(log10IU/mL)	0.864(0.752-0.976)	***<0.001***		76.9	78.1
AST>101(U/L) &HBcrAg<7(log10IU/mL)	0.691(0.538-0.844)	***0.01***		40.0	98.1

AUROC, area under the ROC curve; ALT, alanine aminotransferase; AST, aspartate aminotransferase; HBsAg, hepatitis B surface antigen; HBcrAg, hepatitis B core-related antigen; cccDNA, covalently closed circular DNA.

P values <0.05 are shown in bold.

HBeAg-negative patients with G1, G2, G3 and G4 at baseline were 4(20.0%), 11(55.0%), 5(25.0%) and 0(0.0%), respectively (**Supplementary Figure 3**). All these serum and intrahepatic HBV markers, ALT and AST had no difference between patients with or without significant inflammation (**Supplementary Table 1**).

### Inflammation changes after 60 months of NAs therapy

**Figure 3** shows 59 out of 74 HBeAg-positive patients have undergone liver biopsies after 60 months of NAs therapy, with 94.9% (56/59) of G1 and 5.1% (3/59) of G2. Among the 59 patients, 32 (54.2%) achieved inflammation regression at month 60, including 17 (28.8%) had 1- grade regression, 12 (20.3%) had 2- grade regression, and 3 (5.1%) had 3-grade regression (**Table 4**). No patients had inflammation progression.

**Table 4:** Inflammation changes in HBeAg-positive patients received 60 months of NAs therapy according to Scheuer scoring system.

Inflammation changes at month 60	Baseline inflammation grade (n=74)			
	G1(n=29)	G2(n=25)	G3(n=17)	G4(n=3)
No biopsy(n=16)	3	9	3	0
No change in inflammation(n=26)	26	1	0	0
Improvement in inflammation				
1-grade(n=17)	0	15	2	0
2-grade(n=12)	0	0	12	0
3-grade(n=3)	0	0	0	3

17 out of 20 HBeAg-negative patients have undergone liver biopsies at Month 60, with 94.1% (16/17) of G1 and 5.9% (1/17) of G2 (**Supplementary Figure 3**). Among the 17 patients, 14 (82.4%) achieved inflammation regression, including 10 (58.8%) had 1- grade regression and 4 (23.5%) had 2- grade regression (**Supplementary Table 2**). No patients had inflammation progression.

### Dynamics of serum and intrahepatic HBV markers, ALT and AST during NAs treatment stratified by inflammation change

**Figure 4:**
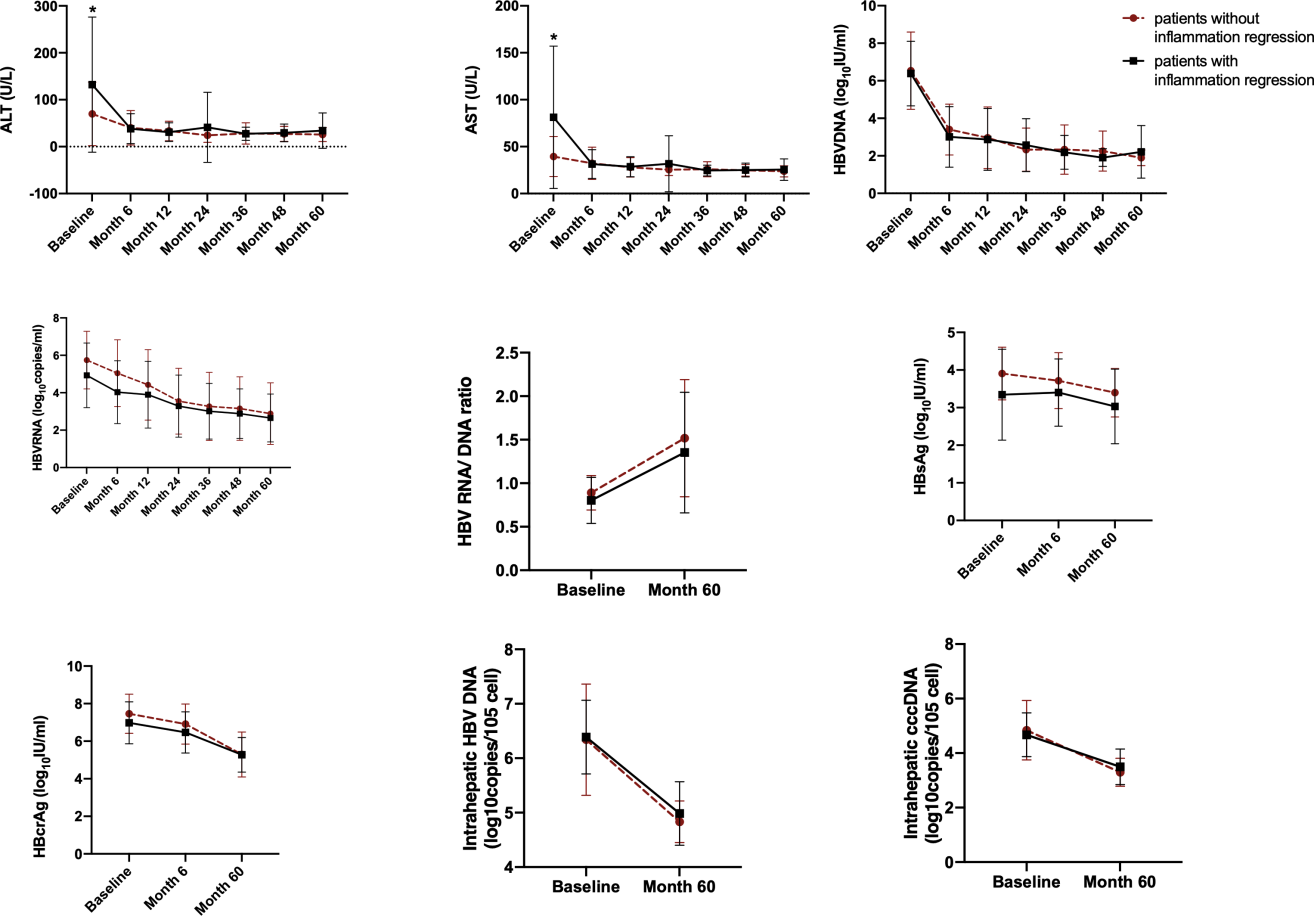
The dynamic changes of HBV markers, ALT and AST in HBeAg-positive CHB patients during NAs treatment. Variables are expressed as means and standard deviations. HBsAg, hepatitis B surface antigen; HBcrAg, hepatitis B core-related antigen; cccDNA, covalently closed circular DNA. *Significant difference between patients with inflammation regression and patients without inflammation regression.

The dynamic changes of serum and intrahepatic HBV markers, ALT and AST from baseline to Month 60 were investigated. All these markers, including serum HBV DNA, HBV RNA, HBsAg and HBcrAg, intrahepatic HBV DNA and cccDNA, as well as ALT and AST, declined in both patients with or without inflammation regression. Moreover, serum ALT, AST and HBV DNA declined most quickly in the first 6th months after initiating NAs therapy (**Figure 4**).

Similar findings were observed in HBeAg-negative patients (**Supplementary Figure 4**).

## Discussion

This study aimed to recognize factors correlated with inflammation grade and capable of diagnosing significant inflammation before NAs treatment. We found that serum HBsAg, HBcrAg, ALT, and AST levels correlated with inflammation grades. The combination of HBsAg and AST exhibited excellent diagnostic ability for significant inflammation in HBeAg-positive CHB patients before NAs treatment. Furthermore, we discovered that after 60 months of NAs treatment, almost all the patients’ liver inflammation ameliorated to G1 according to the Scheuer scoring system.

Hepatic inflammation drives the accumulation of extracellular matrix and causes fibrosis. It is critical to detect inflammation and initiate antiviral treatment timely (Kisseleva and Brenner, 2021). Various guidelines use the ALT level to reflect inflammation; nevertheless, there is evidence that patients with normal ALT have observable liver inflammation, which reveals the limitation of ALT levels in predicting chronic hepatic inflammation (Dufour et al., 2000; Lai et al., 2007; Kumar et al., 2008; Nguyen et al., 2015). Since inflammation eventually drives the development of hepatic fibrosis, potential serum markers that predict hepatic fibrosis may be used to grade inflammatory activity. In this study, we found that serum HBsAg and HBcrAg levels negatively, as well as ALT and AST levels, positively correlated with inflammation grade in HBeAg-positive patients at baseline. While after 60 months of NAs treatment, these correlations disappeared. Consistent with our findings, one previous research showed that HBeAg-positive chronic HBV infection patients with inflammation have a significantly lower HBsAg value than those without inflammation, and HBsAg value was a predictive factor for inflammation (Zeng et al., 2022). Further, Zhang’s paper reported that HBcrAg could predict severe necro-inflammation in both HBeAg-positive and HBeAg-negative patients (Zhang et al., 2016). However, in our study, the relationship between HBV markers and inflammation was not found in HBeAg-negative patients. After 60 months of NAs treatment, intrahepatic HBV DNA and cccDNA correlated with inflammation grade in HBeAg-positive patients, which agrees with a published study that indicated that baseline HBV cccDNA is an independent predictor of liver inflammation (Liang et al., 2016).

The ability of these variables to diagnose significant inflammation in CHB patients before treatment was also explored. And we found that older age, lower serum HBsAg and HBcrAg levels, as well as higher serum ALT and AST levels, were associated with significant inflammation. The ROC analyses revealed that all these variables could diagnose significant inflammation, in which HBsAg had the best performance with an AUROC of 0.784. Furthermore, combining HBsAg and AST improved this classification ability with an AUROC of 0.896.

At baseline, 60.8% of HBeAg-positive and 80.0% of HBeAg-negative patients exhibited inflammation ≥G2. After 60 months of NAs therapy, almost all the patients’ liver inflammation ameliorated to G1, irrespective of the HBeAg status. Therefore, the benefit of antiviral treatment on histology is apparent. Consistent with a previous study (Wang et al., 2019), serum levels of HBV RNA, HBV DNA, HBsAg, HBcrAg, AST and ALT, as well as intrahepatic HBV DNA and cccDNA, declined after initiating antiviral treatment. Moreover, serum ALT, AST and HBV DNA decreased most quickly in the first 6th months.

This study showed that combining ALT and HBsAg offers an attractive alternative to biopsy for assessing inflammation in HBeAg-positive patients and may help make treatment decisions in the clinical setting. Future studies could further explore whether the combination of serum markers could evaluate inflammation in different stages of CHB. This study has several limitations. First, the single-center design and limited sample size may bias the study. Second, this study employed a cohort recruited long ago. The quantification of HBV RNA using cryopreserved serum samples may result in a bias due to the degradation of HBV RNA over time. Besides, only Chinese patients were recruited; thus, the results should be carefully extrapolated to other ethnic groups. Future multi-center studies with a large sample size are needed to confirm the results of this study.

In conclusion, in HBeAg-positive CHB patients, serum HBsAg, HBcrAg, ALT, and AST levels correlated with inflammation grade, and the combination of HBsAg and AST exhibited excellent diagnostic ability for significant inflammation before NAs treatment.

## Data availability statement

The raw data supporting the conclusions of this article will be made available by the authors, without undue reservation.

## Ethics statement

The studies involving human participants were reviewed and approved by the Institutional Review Board of Beijing YouAn Hospital, Capital Medical University (Beijing, China). The patients/participants provided their written informed consent to participate in this study.

## Author contributions

SZ, FL, ZH, ZD, and XC contributed to conception and design of the study. JZ, DB, YW, YR, SL, HL, and YJ organized the database. JZ, DB, and YW performed the statistical analysis. JZ and DB wrote the first draft of the manuscript. YJ, YR, and HF wrote sections of the manuscript. SZ takes responsibility for the integrity of the work as a whole, from inception to published article. All authors contributed to manuscript revision, read, and approved the submitted version.
