# Supplementary material for: Serum HBsAg and HBcrAg is associated with inflammation in HBeAg-positive chronic hepatitis B patients
Source: Front Cell Infect Microbiol. 2023 Mar 31;13:1083912. doi: 10.3389/fcimb.2023.1083912 (PMC10102387; doi:10.3389/fcimb.2023.1083912)
Supplement: Supplementary file 1 [file DataSheet_1.docx]

**Supplementary Materials**

**Methods**

**HBV RNA Assay**

The specially modified super-cis nano-magnetic beads efficiently adsorbed and enriched nucleic acids from 200 µL serum. For DNase I treatment, every reaction mixture comprised 2 µL of DNase I Reaction Buffer (10×), 2 µL of DNAse I (RNase-free), and 16 µL of total nucleic acids. The reaction was carried out at 37°C for 30 min. Next, each mixture was incubated at 75°C for 10 min to inactivate DNase I. Finally, DNase-I-treated HBV RNA was one-step of reverse-transcribed and real-time fluorescent quantitative PCR using the HBV pgRNA high-sensitivity quantitative kit (Sansure Biotech, Changsha, China). Serum HBV RNA levels were measured using SLAN-96P Real-Time PCR Systems (Shanghai Hongshi Medical Technology Co., Ltd, Shanghai, China), with the following amplification profile: an initial denaturation cycle of 1min at 95 °C, an reverse-transcription cycle of 30min at 60 °C, 95 °C for 1 min denaturation, followed by 45 cycles of denaturation for 30 s at 94 °C, annealing for 60 s at 62 °C (the fluorescence signal was collected). The amplification results were automatically analyzed. The LOD of the assay was 200 copies/mL.

**Supplementary Figure1** Correlation of HBV markers, ALT and AST with inflammation grade according to Scheuer scoring system in HBeAg-negative CHB patients at baseline. HBsAg, hepatitis B surface antigen; HBcrAg, hepatitis B core-related antigen.

**Supplementary Figure2** Correlation of HBV markers, ALT and AST with inflammation grade according to Scheuer scoring system in HBeAg-negative CHB patients after 60 months of NAs therapy. HBsAg, hepatitis B surface antigen; HBcrAg, hepatitis B core-related antigen.

**Supplementary Figure3** Inflammation changes after 60 months of NAs therapy in HBeAg-negative CHB patients.

**Supplementary Figure4** The dynamic changes of HBV markers, ALT and AST in HBeAg-negative CHB patients during NAs treatment. Variables are expressed as means and standard deviations. HBsAg, hepatitis B surface antigen; HBcrAg, hepatitis B core-related antigen; cccDNA, covalently closed circular DNA.
