# Supplementary material for: Serum HBsAg and HBcrAg is associated with inflammation in HBeAg-positive chronic hepatitis B patients
Source: Front Cell Infect Microbiol. 2023 Mar 31;13:1083912. doi: 10.3389/fcimb.2023.1083912 (PMC10102387; doi:10.3389/fcimb.2023.1083912)
Supplement: Supplementary file 2 [file Table_1.docx]

Supplementary Table 1 Variables associated with significant inflammation (G ≥ 3 according to the Scheuer scoring system) in HBeAg-negative CHB patients at baseline.

|  | Total (n=20) | Liver inflammation levels at baseline | |  |
| --- | --- | --- | --- | --- |
|  |  | G<3  N= (15) | G≥3  N= (5) | *P* |
| Age (year) | 41.5（24-56） | 42(28-56) | 34(24-45) | 0.19 |
| Male /Female | 16/4 | 12/3 | 4/1 | 1.0 |
| BMI (Kg/m^2^) | 23.8(16.2-31.5) | 24.1(16.2-31.5) | 22.8(20.4-24.1) | 0.13 |
| HBV Genotype † |  |  |  |  |
| C/ others | 5/3 | 3/3 | 2/0 | 0.46 |
| Treatment (n (%)) |  |  |  | 1.0 |
| ETV /ADV | 5/15 | 4/11 | 1/4 |  |
| ALT (U/L) | 102.3(17.2-527.5) | 104.4（18.6-527.5) | 78.9（17.2-183.6) | 0.47 |
| AST (U/L) | 56.2(16.0-200.6) | 56.5（16.0-200.6) | 45.0（22.6-193.7） | 0.84 |
| HBVDNA (log_10_IU/mL) | 4.55(1.70-8.32) | 4.58（1.70-8.04） | 4.37(1.99-8.32) | 0.85 |
| HBVRNA (log_10_copies/mL) | 3.71(1.40-6.10) | 3.75（1.40-6.10） | 3.36（1.40-5.08) | 0.80 |
| HBV RNA/ DNA ratio | 0.69(0.38-1.61) | 0.71(0.38-1.37) | 0.64(0.38-1.61) | 0.98 |
| HBsAg (log_10_IU/mL) | 3.07（1.67-3.63) | 3.04（1.67-3.63） | 3.13（2.90-3.44） | 0.30 |
| HBcrAg (log_10_IU/mL) | 5.29（3.40-7.27） | 4.87（3.56-7.27） | 5.78（3.40-6.03） | 0.76 |
| Intrahepatic HBV DNA  (log10copies/105 cell) | 5.82(3.88-6.98) | 5.77(3.88-6.98) | 5.86(5.56-6.50) | 0.35 |
| Intrahepatic cccDNA  (log10copies/105 cell) | 4.05(3.12-5.09) | 4.20(3.12-5.09) | 4.05(3.61-4.73) | 0.97 |

†Eight patients with available genotype data were analyzed.

Continuous variables are expressed as medians and ranges; categorical variables are expressed as frequencies.

ALT, alanine aminotransferase; AST, aspartate aminotransferase; BMI, Body Mass Index; ETV, Entecavir; ADV, Adefovir dipivoxil; HBsAg, hepatitis B surface antigen; HBcrAg, hepatitis B core-related antigen; HBeAg, hepatitis B e antigen; cccDNA, covalently closed circular DNA.

Supplementary Table 2 Inflammation changes in HBeAg-negative patients received 60 months of NAs therapy according to Scheuer scoring system.

| Inflammation changes at month 60 | Baseline inflammation grade (n=20) | | | |
| --- | --- | --- | --- | --- |
|  | G1(n=4） | G2(n=11) | G3(n=5) | G4(n=0) |
| No biopsy(n=6) | 1 | 2 | 3 | 0 |
| No change in inflammation(n=3) | 3 | 0 | 0 | 0 |
| Improvement in inflammation |  |  |  |  |
| 1-grade(n=10) | 0 | 9 | 1 | 0 |
| 2-grade(n=4) | 0 | 0 | 4 | 0 |
| 3-grade(n=0) | 0 | 0 | 0 | 0 |
