# Supplementary figures and images for: Serum HBsAg and HBcrAg is associated with inflammation in HBeAg-positive chronic hepatitis B patients
Source: Front Cell Infect Microbiol. 2023 Mar 31;13:1083912. doi: 10.3389/fcimb.2023.1083912 (PMC10102387; doi:10.3389/fcimb.2023.1083912)

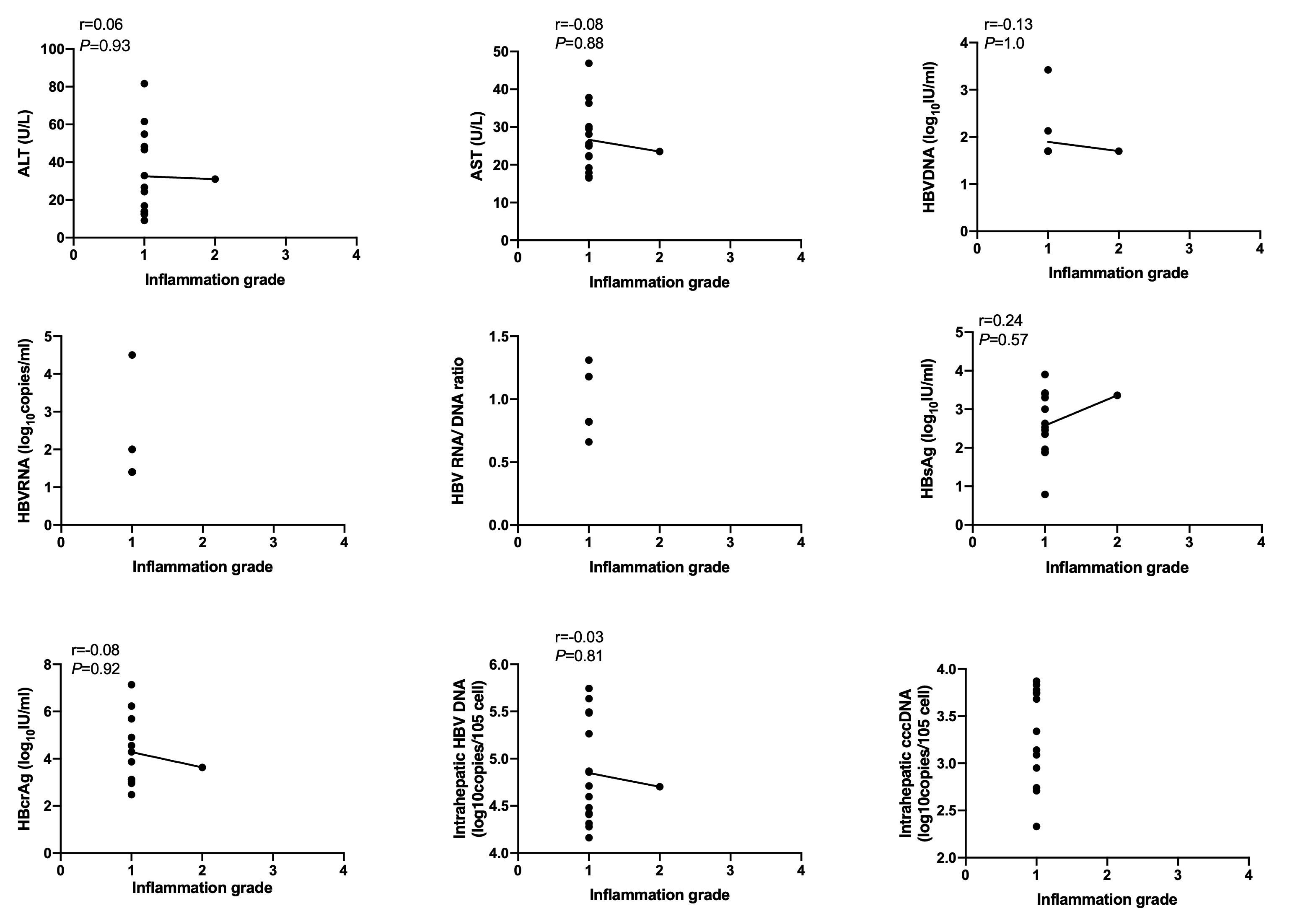

Supplement: Supplementary file 3 [file Image_1.tiff]

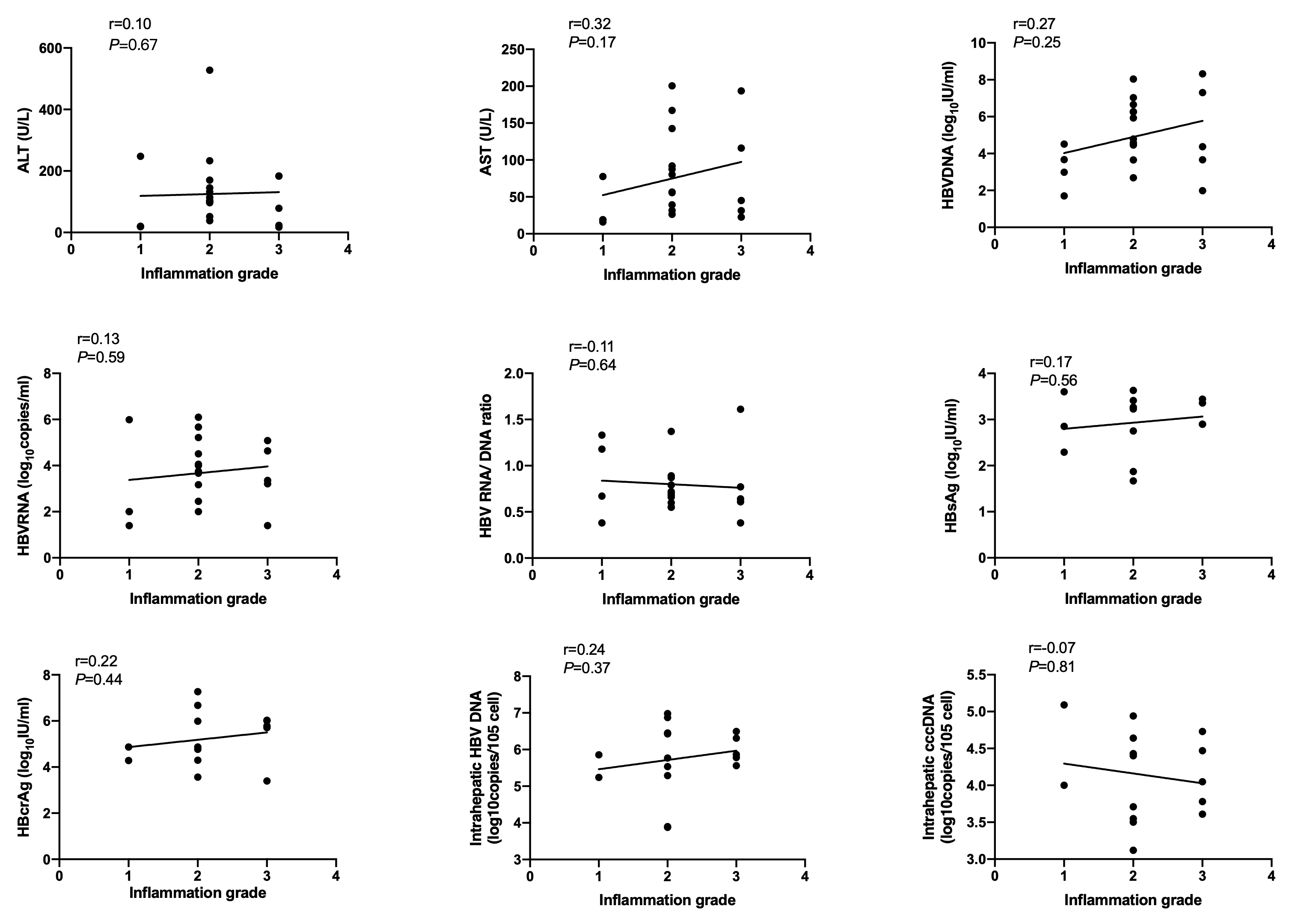

Supplement: Supplementary file 4 [file Image_2.tiff]

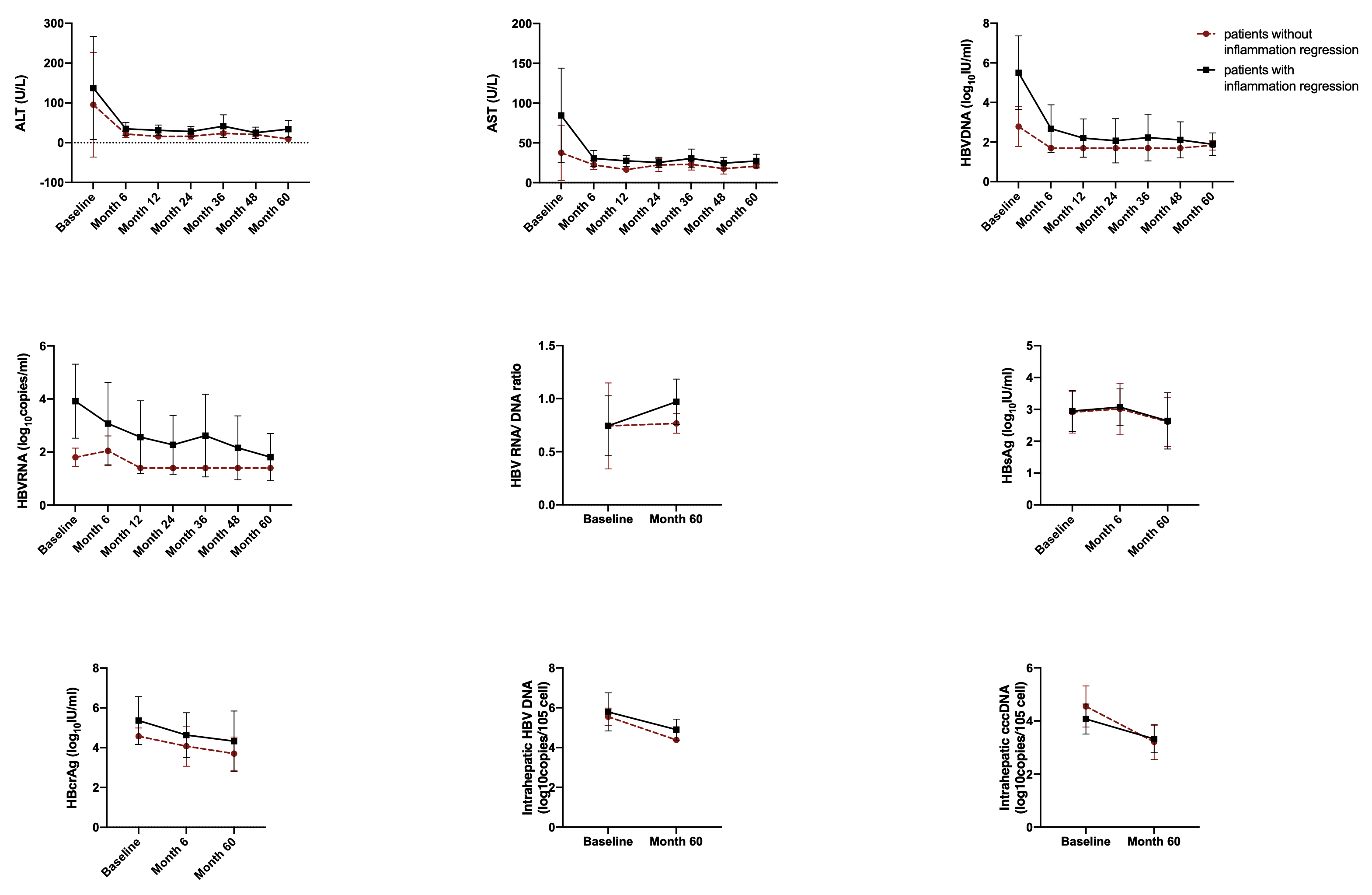

Supplement: Supplementary file 5 [file Image_3.tiff]

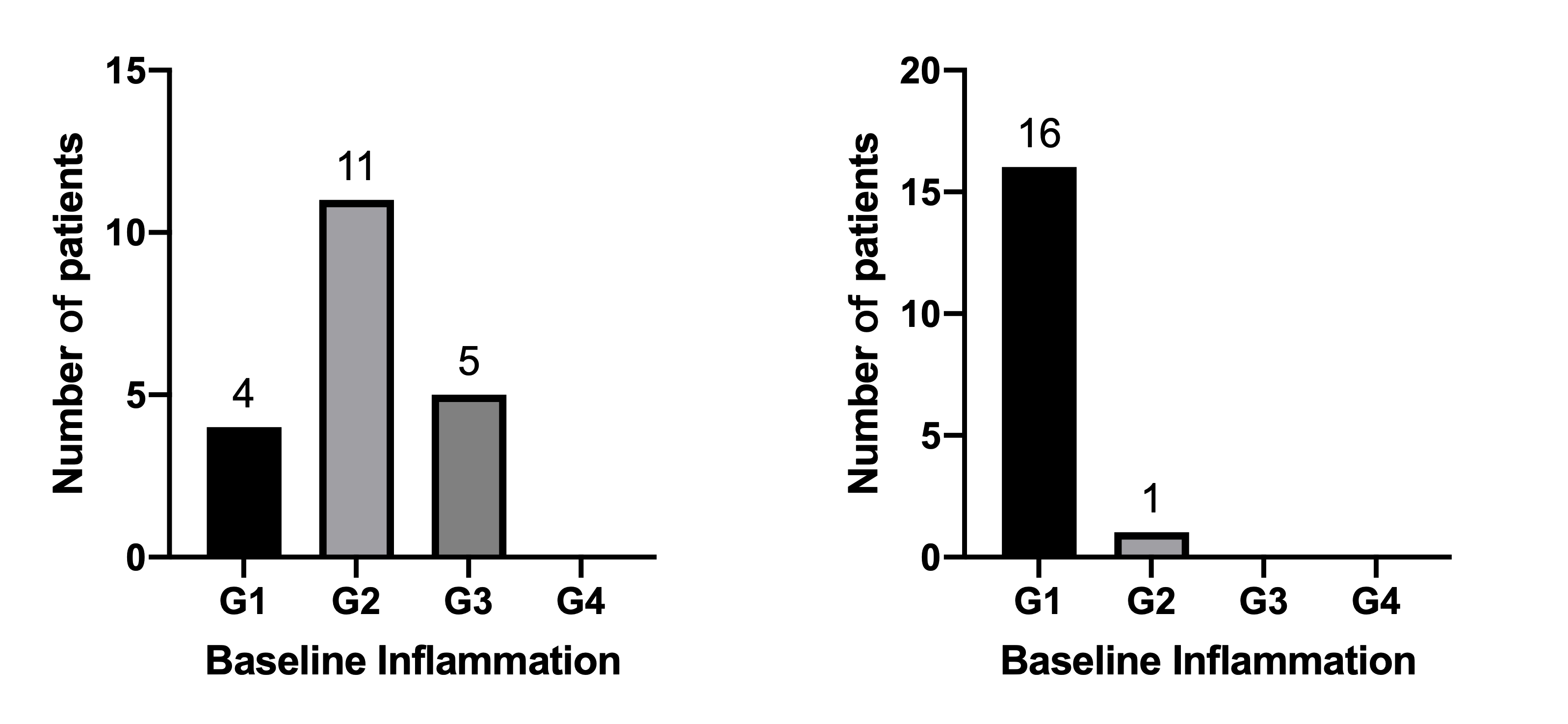

Supplement: Supplementary file 6 [file Image_4.tiff]
